# Profiling the Effect of Micronutrient Levels on Vital Cardiovascular Markers

**DOI:** 10.7759/cureus.78268

**Published:** 2025-01-30

**Authors:** Hari K. Krishnamurthy, Swarnkumar Reddy, Vasanth Jayaraman, Karthik Krishna, Qi Song, Tianhao Wang, Kang Bei, John J Rajasekaran

**Affiliations:** 1 Research and Development, Vibrant Sciences LLC, San Carlos, USA; 2 Clinical Laboratory, Vibrant America LLC, Santa Clara, USA; 3 Clinical Laboratory, Vibrant America LLC, San Carlos, USA

**Keywords:** amino acids, branched-chain amino acids, cardiovascular diseases, lipids, micronutrients, vitamins

## Abstract

Introduction

Cardiovascular diseases (CVDs) remain the leading cause of mortality globally. The role of micronutrients in maintaining cardiovascular health has gained increasing attention, as deficiencies or imbalances in vitamins, minerals, and amino acids may influence the risk and progression of CVDs. This study aimed to evaluate the relationship between serum micronutrient levels and critical lipid and lipoprotein markers indicative of cardiovascular health.

Materials and methods

A retrospective analysis was conducted on 358 individuals who underwent testing for the Cardio Health and Micronutrients Panel at Vibrant America Clinical Laboratory. The participants were divided into three groups based on their serum lipid and lipoprotein concentrations: ‘Low’, ‘Normal’, and ‘High’. The levels of vitamins (A, D, E, and K), minerals (zinc, iron, calcium, and magnesium), and amino acids (leucine, isoleucine, and valine) were measured, and their correlation with cardiovascular markers such as cholesterol, LDL, HDL, and Apo B was analyzed using Pearson’s correlation.

Results

The study found significant associations between micronutrient levels and cardiovascular markers. Vitamins D, E, and K and minerals like zinc, calcium, and magnesium showed positive correlations with lipid markers. Asparagine was negatively correlated with cholesterol and LDL, while amino acids such as isoleucine and valine negatively correlated with HDL but showed a positive association with LDL and Apo B. Fat-soluble vitamins demonstrated strong positive associations with total cholesterol and triglycerides.

Conclusion

These findings suggest that certain micronutrients play a critical role in regulating lipid profiles and overall cardiovascular health. Further studies are necessary to explore the therapeutic potential of micronutrient supplementation in preventing or managing CVDs.

## Introduction

Micronutrients are the most essential nutrients for a whole range of physiological functioning of a living system. Dietary deficiency of micronutrients leads to pathogenesis, progression, and morbidity in various critical clinical disorders including cardiovascular diseases (CVDs) [1]. The pathobiology of CVDs remains complex and it is generally regarded to be a result of various genetic predispositions interacting with environmental factors. More than 40% of CVDs are related to nutritional factors and more than 90% of such cases were attributed to preventable factors with nutrition as a major determinant. Atherosclerosis and hypertension are the predominant chronic CVDs that are profoundly preventable with a multi-component strategy including nutrient, diet, and lifestyle interventions [1]. Atherosclerosis, characterized by the chronic inflammation of arteries affecting the function of the heart, results from the deposition of fats, cholesterol, and other substances on the inner lining of the artery eventually resulting in CVDs or heart failure [2]. It is estimated that ≥ 2% of the adult population has atherosclerosis which tends to increase up to 10% or higher in people above 70 years and continues to increase with the age of the population [3]. Hypertension is another predominant CVD, which is clinically referred to as a chronic sustained increase in arterial blood pressure. Hypertension is the most widespread CVD with more than 35% of the population affected globally. A report from the World Health Organization stated this might increase beyond 50% in 2025 [4]. Due to cardiac disease being a complex multifunctional etiology, its precise mechanism of pathophysiology remains unclear in both atherosclerosis and hypertension. Atherosclerosis is characterized by the impaired flow of blood and hypertension is characterized by a chronic increase in blood pressure, both these conditions result in abnormal cardiac functioning and vascular damage [5].

CVDs include a wide spectrum of risk factors such as genetic, behavioral, and environmental factors. In most cases, CVDs commonly result from the interplay of multiple risk factors; common classical risk factors include age, any pre-existing medical illness such as diabetes, hypertension, predisposition genetics, or behavioral factors like smoking. Among these risk factors, environmental factors are the most easily modifiable and play a pivotal role in cardiovascular health. The environmental factors are the least investigated risk factors and are noted as the most preventable risk factors of CVDs. Environmental factors include the intake of micronutrients such as vitamins, minerals, and amino acids. The micronutrients include organic compounds such as Vit A, Vit C, Vit D, Vit E, and Vit K, dietary minerals including calcium, sodium, potassium, magnesium, etc, and trace elements like iron, chromium, copper, selenium, and zinc. Several clinical reports have shown a significant correlation between the deficiency of one or more micronutrients and CVDs. However, those reports were limited by the diversity of the micronutrients studied. For instance, Rai et al. [6] evaluated the effect of two micronutrients copper and zinc on lipid profiles in healthy adults, and another study by Islam et al. [5] reported that no significant impact was observed by the consumption of vitamin D with calcium on serum lipids. The current study aimed to broadly investigate the correlation between cardiovascular markers and the levels of 32 micronutrients. We also attempted to hypothesize the pathophysiology of micronutrient deficiency through which the cardiobiomarkers could be affected.

This article was previously posted to the medRxiv preprint server on April 25, 2023.

## Materials and methods

Study population

This study involved a retrospective analysis of de-identified data from 358 participants, with a mean age of 48 ± 15 years, who underwent cardiovascular and micronutrient testing at the Vibrant America Clinical Laboratory. The participants represented a general, free-living population without clinical indications of significant abnormalities. The research was conducted in compliance with ethical standards and was exempt from formal ethical review by the Western IRB (IRB # 1-1288754-1, Washington, USA) due to the retrospective nature of the study and the use of de-identified samples.

Participants were categorized based on their serum lipid and lipoprotein levels. These classifications included normal and altered levels of total cholesterol, LDL, HDL, triglycerides, apolipoproteins (Apo A1, Apo B), and lipoprotein (a) [Lp(a)], which are key markers in cardiovascular health.

Cardiovascular panel

Blood samples were collected, processed to separate the serum, and then analyzed using a comprehensive cardiovascular panel. The panel included lipid measurements such as total cholesterol, LDL cholesterol, HDL cholesterol, and triglycerides, in addition to apolipoproteins (Apo A1 and Apo B) and lipoprotein (a) [Lp(a)] as specific markers.

Total Cholesterol

Total cholesterol was quantified using the cholesterol dehydrogenase method on a Beckman Coulter AU680 analyzer (Beckman Coulter, CA, USA).

LDL, HDL, and Triglycerides

These were measured via an enzymatic-colorimetric method, also on the Beckman Coulter AU680 analyzer.

Apo A1, Apo B, and Lp(a)

Apo A1, Apo B, and Lp(a) were determined using a particle-enhanced immunoturbidimetric assay conducted with Roche Cobas 6000 c 501 analyzers (Roche Cobas, CA, USA).

These cardiovascular markers were selected based on their established role in assessing cardiovascular risk and health, offering a broad profile of both traditional and emerging risk factors.

Micronutrient panel

Micronutrient levels were measured from serum samples using advanced technologies, providing precise quantitative data.

Vitamins and Minerals

Vitamins and minerals were analyzed using Waters TQ-XS Tandem mass spectrometry coupled with LCMS (liquid chromatography mass spectrometry), Waters GC-MS (gas chromatography-mass spectrometry; Waters, CA, USA), and PerkinElmer NexION ICP-MS (inductively coupled plasma mass spectrometry; PerkinElmer, CA, USA).

These methodologies ensure high sensitivity and specificity for a range of essential micronutrients, including vitamins, trace elements, and minerals. Standardized protocols were followed to maintain accuracy and repeatability across all measurements.

Data collection

Data were collected from laboratory databases, including patient demographic information (age, sex) and laboratory results for both the cardiovascular and micronutrient panels. De-identification of the data was performed to ensure confidentiality, in compliance with relevant regulations.

Statistical analysis

Clinical data were subjected to retrospective analysis from de-identified subjects using Java for Windows version 1.8.161 (Oracle Corporation, Austin, USA). A non-parametric Mann-Whitney U test was used to compare the micronutrients with normal and altered lipid and lipoprotein concentrations. Pearson’s correlation was carried out to analyze the univariant relationship between serum lipids, lipoproteins, and micronutrients with significance set at p<0.05. All statistical analyses were performed using GraphPad Prism Version 7.00 (GraphPad Software, San Diego, CA) and a descriptive statistic was used to define the continuous variables (mean ± SD, median, minimum, and maximum).

## Results

The study group comprised 165 (46.1%) men and 193 (53.9%) women with a mean age group of 47.9 ± 15.4. Table 1 shows the descriptive statistics of the lipid and lipoprotein profiles of 358 subjects. The study aimed to evaluate the significant relationship of the wide range of micronutrients including vitamins, dietary minerals, trace elements, and organic compounds with cholesterol (total, LDL, HDL) and triglycerides as well as apo A, apo B, and Lp (a) levels.

**Table 1:** Serum Lipid and Lipoprotein Levels of Candidates

Cardiovascular marker	n=358	Frequency N (%)	Mean ± SD
	Male	165 (46.1)	48.1 ± 16.6
	Female	193 (53.9)	47.8 ± 14.4
Cholesterol ≤199 mg/dL	Low	-	-
	Normal	296 (82.6)	183.3 ± 29.9
	High	62 (17.3)	265.5 ± 26.6
Low-density lipoprotein ≤99 mg/dL	Low	-	-
	Normal	212 (59.2)	98.3 ± 21.1
	High	146 (40.7)	163.8 ± 27.7
High-density lipoprotein ≥56 mg/dL	Low	36 (10)	35.1 ± 5.4
	Normal	320 (89.3)	59.5 ± 15.6
	High	02 (0.5)	-
Triglyceride ≤149 mg/dL	Low	-	-
	Normal	323 (90.2)	87.6 ± 36.0
	High	35 (9.7)	314.2 ± 168.4
Apolipoprotein A1 ≥120 mg/dL	Low	47 (13.1)	116.8 ± 17.0
	Normal	311 (86.8)	169.0 ± 30.4
	High	2 (0.5)	-
Apolipoprotein B ≤89 mg/dL	Low	-	-
	Normal	270 (75.4)	88.9 ± 18.0
	High	88 (24.6)	144.0 ± 30.8
Lipoprotein (a) ≥30 mg/dL (N= 241)	Low	-	-
	Normal	157 (65.1)	14.1 ± 6.4
	High	84 (34.8)	69.4 ± 34.9

The subjects were classified based on the levels of serum lipids and lipoproteins. In the case of total cholesterol (TC), LDL, triglycerides, and Apo B, the subjects were categorized based on lipid concentrations higher than the reference range and subjects within the reference range while in the case of HDL and apo A, the subjects were divided into lipid concentrations lower than the reference range and subjects within the reference range. A significant statistical relationship was observed between various micronutrients and serum lipid and lipoprotein concentrations as shown in Table 2. An increase in the serum levels of Vit E, Vit D3, and Mg was found to be statistically significant with increased levels of serum cholesterol and LDL (Table 2 and Table 3). A decrease in serum levels of HDL was significantly affected by the serum levels of Vit D 25(OH), asparagine, glutamine, and serine (Table 4). A high level of serum triglycerides is significantly associated with increased levels of various micronutrients including Vit A, Vit D 25(OH), Vit E, and Vit K1 and amino acids such as glutamine, serine, isoleucine, valine, and leucine (Table 5).

**Table 2:** Micronutrient Comparison for Cholesterol Levels

Cholesterol					
	Greater than the reference range (n=62)		Within range (n=296)		P (p<0.05)
Vitamin E	16±5.7	14.4 (7.9-29.4)	12.9±4.2	11.9 (5.3-30.7)	0.0001
Vitamin D3	1.1±0.2	1.1 (0.4-1.7)	0.8±0.2	0.8 (0.3-1.6)	0.0001
Vitamin K1	2.1±1.5	1.5 (0.1-7.3)	1.4±1.3	1.2 (0.05-13.9)	0.0003
Calcium	9.8±0.3	9.8(9-10.6)	9.6±0.4	9.6 (8.5-11.2)	0.0001
Magnesium	2.2±0.1	2.2 (1.9-2.6)	2.1±0.1	2.1 (1.0-2.6)	0.0001

**Table 3:** Micronutrient Comparison for Low-Density Lipoprotein (LDL) Levels

Low-density lipoprotein					
	Greater than the reference range (n=146)		Within range (n=212)		P (p<0.05)
Vitamin E	14.8±4.8	13.6(7.3-29.4)	12.3±4.2	11.2(5.3-30.7)	0.0001
Vitamin D3	1.04±0.26	1.02(0.44-1.78)	0.84±0.81	0.81(0.32-1.63)	0.0001
Iron	108±33.3	108.1(35.5-201.8)	99.1±41.4	91.4(25.3-238.9)	0.0024
Magnesium	2.23±0.16	2.2(1.5-2.66)	2.16±0.19	2.1(1.0-2.6)	0.0002

**Table 4:** Micronutrient Comparison for High-Density Lipoprotein (HDL) Levels

High-density lipoprotein					
	Less than the reference range (n=36)		Within range (n=301)		P (p<0.05)
Vitamin D 25 OH	40.7±24.9	33.6(12.4-136)	48.3±22.8	41.7(8.8-133)	0.0091
Asparagine	105.6±42.6	110.1(34.1-220.6)	53.9±11.6	52.9(28.1-108.0)	0.0001
Glutamine	212.5±164.3	124.6(61.0-671.4)	508.6±80.7	510.9(202.2-752.4)	0.0001
Serine	128.0±36.2	124.7(61.0-282.9)	149.4±34.2	145.6(59.6-274.5)	0.0001

**Table 5:** Micronutrient Comparison for Triglyceride Levels

Triglycerides					
	Greater than the reference range (n=36)		Within range (n=323)		P (p<0.05)
Vitamin E	20.1±17	16.7(8.5-118.7)	12.9±4.3	11.9(5.3-29.4)	0.0001
Vitamin A	88.4±27.3	82(52.8-151.9)	75.8±23.3	72.1(34.1-151.5)	0.0061
Vitamin K1	3.14±2.7	2.3(0.22-13.9)	1.42±0.99	1.22(0.05-7.32)	0.0001
Vitamin D25 OH	33.9±13.6	30.7(15.4-71.2)	48.6±23.2	41.8(8.8-136)	0.0001
Glutamine	463.7±107.6	472.9(118.7-608.3)	508.4±78.7	507.6(202.2-752)	0.0290
Serine	136.5±32.8	130.6(76.0-198.5)	149.6±34.7	145.6(59.6-282.9)	0.0368
Carnitine	32.6±16.5	31.5(11.6-118.7)	26.5±7.2	26.6(4.1-50.5)	0.0014
Isoleucine	84.0±27.5	84.2(35.7-142.7)	67.1±19.1	64.6(24.0-139.7)	0.0002
Valine	275.0±63.3	281.1(175.3-442.4)	241.7±52.8	233.6(134.9-442.7)	0.0020
Leucine	179.9±36.8	182.7(113.7-233.5)	161.7±34.1	157.4(79.7-249.2)	0.0033

Decreased levels of serum Apo A are significantly associated with decreased serum levels of Vit E, Vit D3, asparagine, and minerals such as calcium, zinc, and iron (Table 6). The elevated levels of Apo B associated with increased serum LDL, and non-HDL fats are found to be statistically significant with all classes of micronutrients including vitamins, minerals, amino acids, and trace elements (Table 7). 

**Table 6:** Micronutrient Comparison for Apolipoprotein A Levels

Apolipoprotein A					
	Less than the reference range (n=47)		Within range (n=323)		P (p<0.05)
Vitamin E	12.2±4.8	10.9(4.0-23.4)	13.5±4.7	12.4(5.6-30.7)	0.0369
Vitamin D3	0.84±0.24	0.81(0.41-1.5)	0.94±0.28	0.91(0.32-2.19)	0.0198
Asparagine	51.0±10.6	49.4(34.1-79.1)	54.5±12.1	53.3(28.1-113.0)	0.0444
Calcium	9.4±0.3	9.5(8.7-10.4)	9.6±0.39	9.6(8.5-11.1)	0.0022
Zinc	0.69±0.19	0.64(0.45-1.50)	0.71±0.15	0.69(0.39-2.23)	0.0315
Iron	85.6±36.6	78.5(25.3-182.4)	105.5±38.4	101.6(33.2-238.9)	0.0015

**Table 7:** Micronutrient Comparison for Apolipoprotein B Levels

Apolipoprotein B					
	Greater than the reference range (n=87)		Within range (n=270)		P (p<0.05)
Vitamin E	16.0±5.5	15.3(7.3-29.0)	12.5±4.1	11.7(4.0-30.7)	0.0001
Vitamin A	82.4±23.7	77.7(34.1-145.8)	75.8±24.2	72.1(37.1-151.9)	0.0096
Vitamin D3	0.87±0.24	0.83(0.32-1.6)	1.09±0.30	1.08(0.44-2.19)	0.0001
Vitamin K1	1.5±1.3	1.2(0.05-13.9)	1.8±1.4	1.4(0.10-7.3)	0.0416
Selenium	146.4±24.0	144.8(93.4-210.8)	142.3±30.3	139(95.7-360.4)	0.0451
Potassium	4.5±0.35	4.5(3.6-5.6)	4.5±0.40	4.49(3.1-6.22)	0.0377
Calcium	9.7±0.3	9.7(8.7-10.6)	9.6±0.3	9.6(8.5-11.1)	0.0055
Zinc	0.73±0.11	0.72(0.39-1.0)	0.70±0.17	0.68(0.44-2.23)	0.0058
Iron	109.3±34.2	107.7(45.6-201.3)	100.8±39.9	97.7(25.3-238.9)	0.0239
Magnesium	2.2±0.1	2.2(1.9-2.9)	2.1±0.19	2.1(1.0-2.6)	0.0002
Isoleucine	72.7±19.9	70.7(34.0-121.4)	67.6±20.7	64.2(24.0-142.7)	0.0138
Valine	261.9±55.2	262.7(146.5-404.0)	240.2±54.2	232.1(134.9)	0.0004
Leucine	172±34.8	168.8(113.7-248.2)	160.5±34.2	157.4(79.7-249.2)	0.0138
Serine	140.6±33.0	135.4(75.0-209.7)	151.1±34.7	147.2(59.6-282.9)	0.0189

Quantitative analysis by Pearson’s correlation showed that serum concentration of cholesterol had a strong positive correlation with serum levels of Vit E (r=0.4086, p<0.0001), Vit D3 (r=0.5125, p<0.001), and Vit K1 (r=0.2334, p<0.0001) The cholesterol levels are also considerably correlated with Vit A and iron (<0.05). The serum levels of cholesterol were found to have a significantly negative correlation with folate and asparagine. LDL was found to have a significant positive correlation with serum levels of Vit E (r=0.3398, p<0.0001) and Vit D3 (r=0.4774, p<0.0001).

**Figure 1:**
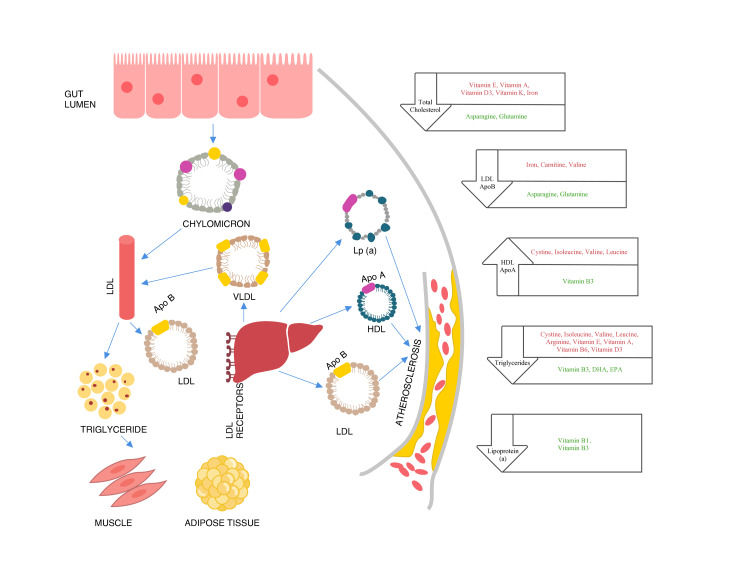
Role of Micronutrients in Regulating Serum Lipids Micronutrients in green font reduce the risk by modulating the particular component of the lipid panel. Micronutrients in red font increase the risk by modulating the particular component of the lipid panel. Image Credits: Hari Krishnan Krishnamurthy and Swarnkumar Reddy

**Figure 2:**
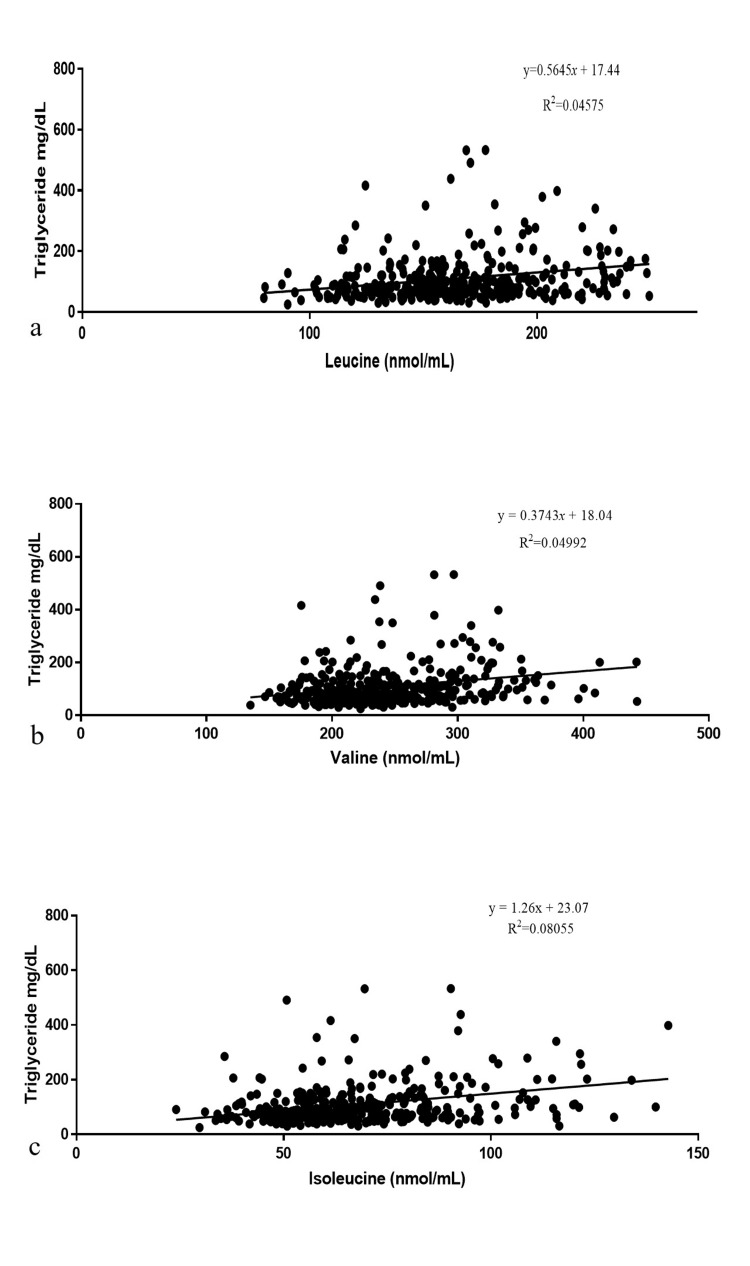
Correlation between Serum Triglycerides and Serum BCAAs a. Leucine, b. Valine c. Isoleucine The image shows three scatter plots depicting the relationship between triglyceride levels (mg/dL) on the y-axis and three different amino acids (nmol/mL) on the x-axis. BCAAs: Branched-chain amino acids

Pearson’s correlation was carried out to analyze the univariant relationship between serum lipids, lipoproteins, and micronutrients with significance set at p<0.05. Significant results are shown in Table 8. The directionality of the variation of micronutrients is represented in Figure 1. It was interesting to note the significant association of branched-chain amino acids (BCAAs) with several markers in the cardiovascular panel. Increased levels of BCAAs, leucine, isoleucine, and valine, showed significant association with decreased HDL levels and increased triglyceride levels as shown in Figure 2. Increased levels of valine were shown to have a significant association with increased LDL and Apo B levels. These associations show a significant association between BCAAs and lipid dysregulation. Additionally, amino acids asparagine and glutamine were found to be inversely correlated with serum LDL. A significant inverse correlation was observed between glutamine and serine with serum levels of Apo B.

**Table 8:** Pearson Correlation of Micronutrients with Serum Lipids and Lipoproteins

	r	p
Cholesterol		
Vitamin E	0.4086	<0.0001
Vitamin A	0.1471	0.0053
Vitamin D3	0.5125	<0.0001
Vitamin K1	0.2334	<0.0001
Vitamin B9	-0.104	0.0493
Iron	0.1067	0.0437
Asparagine	-0.125	0.018
Low-density lipoprotein		
Vitamin D3	0.4774	<0.0001
Iron	0.1199	0.0233
Asparagine	-0.1217	0.0213
Glutamine	-0.1074	0.0423
Carnitine	0.1446	0.0061
Valine	0.1471	0.0053
High-density lipoprotein		
Cysteine	-0.2289	<0.0001
Isoleucine	-0.2298	<0.0001
Valine	-0.2317	<0.0001
Leucine	-0.2405	<0.0001
Triglycerides		
Vitamin E	0.3832	<0.0001
Vitamin A	0.2278	<0.0001
Vitamin B6	-0.1057	0.0457
Vitamin D3	0.1342	0.011
Vitamin K1	0.5732	<0.0001
Cysteine	0.1626	0.002
Isoleucine	0.2838	<0.0001
Valine	0.2234	<0.0001
Leucine	0.2139	<0.0001
Arginine	0.1363	0.0098
Apolipoprotein B		
Glutamine	-0.1388	0.0086
Serine	-0.1269	0.0165
Valine	0.1259	0.0173
Lipoprotein (a)		
Vitamin B1	-0.1818	0.0046

## Discussion

Micronutrient deficiency is a major cause of the pathogenesis, progression, mortality, and morbidity in developing various chronic health issues including CVDs. It is regarded as a vital component of the socio-economic development of a society and hence effective strategies have been framed to address deficiencies at the population level by adding emphasis on dietary improvement, supplementation, food fortification, and global public health control. Understanding the role of individual micronutrients and proper intake of essential micronutrients plays a vital role in preventing micronutrient-related diseases. Micronutrient deficiency due to malnutrition can be prevented by the above-mentioned strategies. However in the context of the current study, the knowledge of the role of individual micronutrients and timely dietary supplementation of micronutrients plays a crucial role in improving physiological functions [7]. Micronutrients such as vitamins, minerals, and amino acids are known to improve cardiovascular health by regulating the serum levels of lipids and lipoproteins. However, the association of micronutrients with cardiovascular markers remains unclear [8]. The present study details the vital micronutrients with significant association with primary cardiovascular markers such as cholesterol, LDL, HDL, triglycerides, Apo A, Apo B, and Lp(a). The study specifies a wide range of micronutrients which includes 13 vitamins, nine minerals, and eight amino acids.

The results of the present study signify a strong positive correlation with high significance between fat-soluble vitamins Vit A, Vit D3, Vit E, and Vit K1 with cholesterol and triglycerides. Few studies have discussed the association of fat-soluble vitamins with lipid profile, Piran et al. [9] attempted to study the association of fat-soluble vitamins A, E, and D. They reported a correlation only between vitamin E and lipid profile, they also suggested the supplementation of vitamin E for overweight subjects to decrease LDL-C levels. Several substantial shreds of evidence have proved that significant changes in mean serum retinol levels resulting from dietary intake or when supplemented to reduce the risk of cancer alter lipid metabolism by increasing circulating levels of triglycerides and cholesterol. Pastorino et al. [10] have stated retinol-induced liver damage by abnormal elevation of alkaline phosphatase with overconsumption of vitamin A. Several reports have demonstrated the association of vitamin D with serum lipid profiles, a study by Dibaba [11] reported an inverse correlation where the supplementation of vitamin D resulted in reducing serum cholesterol, LDL, and triglycerides, but they reported no association between vitamin D and HDL. A randomized trial by Barbarawi et al. [12] observed no association between vitamin D supplementation and reduced risk of major adverse cardiovascular events, myocardial infarction, stroke, etc. In the present result, vitamin D was found to have a positive correlation with cholesterol and triglycerides. As stated by Barbarawi et al. [12], the present result also showed no association with serum HDL. At optimal levels of serum concentrations, vitamin D might be beneficial by reducing cholesterol, LDL, and triglycerides. High doses of Vit D supplements result in irregular heartbeat and can also raise blood calcium levels which lead to heart failure [13]. An increase in vitamin E might interfere with blood clotting by inhibiting platelet aggregation and vitamin K-mediated clotting factors which might lead to hemorrhagic stroke (Natural Medicines Comprehensive Database). Vitamin E below the reference range results in acute heart muscle damage. Vitamins B9 (folate) were found to have a negative correlation with cholesterol and Vit D3 was found to have a negative correlation with serum LDL. Interestingly, no vitamins exhibited any significant correlation with HDL and Apo A. The current results signify that an overdose of fat-soluble vitamins could result in adverse health effects by modulating serological biomarkers. Water-soluble vitamins tend to accumulate less in tissues as they are readily soluble in water and excreted [14]. Among all the analyzed minerals, iron was found to be positively correlated with serum levels of cholesterol (r=0.1067, p<0.0437) and LDL (r=0.1199, p<0.0233).

Numerous studies have demonstrated the significance of micronutrients in cardiovascular health. For instance, Ma et al. [15] suggested that the deficiency in levels of trace minerals such as zinc, copper, iron, and selenium is directly associated with CVDs, and supplementation of these trace elements can prevent CVDs. A review by Panchal et al. [16] has demonstrated the importance of selenium, vanadium, and chromium in improving metabolic syndrome. Li et al. [17] in their study on plasma metabolomics stated that plasma metabolites and serum micronutrient levels can be used to predict the risk of CVDs. Extensive research has increased the understanding of the association between micronutrients and cardiac health. Micronutrients such as vitamins and minerals are widely investigated, whereas amino acids are hardly investigated. A recent report by Tharrey et al. [18] has detailed the association between amino acid intake and cardiovascular mortality and they also reported the negative effect of a few amino acids on cardiac health. The intake of protein and peptide-based health supplements has increased in recent years which could result in increased levels of non-essential amino acids, the effect of this on lipid and lipoprotein levels was analyzed in our study.

Levels of a few vital minerals such as magnesium, potassium, calcium, and zinc have also been known to influence cardiac health. For instance, deficiency in levels of magnesium and potassium results in elevated blood pressure. Calcium levels need to be at an optimum since the deficiency causes pulmonary hypertension, whereas elevated serum calcium might result in cardiovascular risk due to vascular calcification. Insufficient vitamin K2 and vitamin D results in failure to direct calcium away from blood vessels resulting in vascular calcification. Studies have reported vascular calcification due to unregulated calcium resulting from supplementation without adequate vitamin K2. 

To our knowledge, the present study is the first to report on the association of various amino acids with serum lipid and lipoprotein components. The study observed a statistical significance between various amino acids and serum levels of lipids and lipoproteins as represented in Figure 1. Increased levels of asparagine and glutamine were shown to decrease the levels of LDL. Deprivation of asparagine and glutamine results in lethal effects on endothelial cells (ECs). The supplementation of these non-essential amino acids can assist in the recovery of ECs, can also fight against nitrification stress, and aid in angiogenesis. A previous report by Luo et al. [19] on the interactive effects of asparagine and aspartate stated that the subjects with type 2 diabetes were observed to have low levels of HDL-C and reported a negative correlation between serum levels of asparagine and HDL-C. Serum levels of glutamine and serine were found to have no influence on plasma lipids and also regular dietary supplementation of these amino acids has various clinical advantages [20]. A study by Mansour et al. [21] demonstrated the advantage of glutamine supplementation as it remarkably reduced the total cholesterol and blood pressure in patients with type 2 diabetes. They also proposed that supplementation of glutamine can be an effective pharmaconutrient in controlling diabetes, obesity, and other chronic metabolic disorders. Similarly, adequate serine supplementation could help increase antioxidant and anti-fatty streak (lesion in the development of atherosclerosis) activity [22]. The increase in the serum levels of triglycerides is significantly associated with low serum levels of amino acids such as glutamine, serine, leucine, isoleucine, and valine. An increase in the serum levels of triglycerides results in atherosclerosis which leads to high chances of stroke and other related heart diseases [23]. A significant positive association was observed between elevated levels of Apo B and low serum levels of amino acids such as serine and isoleucine. Serum levels of Apo B above adequate levels are associated with the accumulation of LDL-C and non-HCL-C which are associated with various CVDs.

The present study shows an interesting correlation between cardiovascular markers and BCAAs. BCAA leucine, isoleucine, and valine are nonpolar and hydrophobic and are vital nitrogen sources for the synthesis of glutamine and alanine. Several epidemiological studies have proved the association between BCAAs and increased risk of type 2 diabetes [24]. The increase in the levels of BCAAs increases the oxidation of BCAAs in muscles which inhibits the fatty acid oxidation and results in blunted insulin signaling. Diabetes and CVDs are the major causes of morbidity and mortality. A report by Magnusson et al. [24] identified BCAA as a strong predictor of CVD development and also as an early marker for the association between diabetes and CVDs. Another study by Tobias et al. [25] proved that circulating BCAA is a strong predictor of type 2 diabetes. The quantitative analysis of BCAAs, leucine, isoleucine, and valine, with the cardiovascular markers showed a clear association. A strong negative correlation between HDL and BCAAs and a strong positive correlation between triglycerides and BCAAs (Table 3). This signifies that the increase in the serum level of BCAAs decreases the HDL and increases the triglycerides. In the case of LDL and Apo B, a positive correlation was observed with serum level of valine which signifies the increase in the serum levels of valine also increases the levels of LDL and Apo B which could result in CVDs.

The present results identify a few micronutrients with the possibility of being pathogenic, for instance, the positive correlation between vitamin D and serum cholesterol and LDL. The same association has been previously reported by numerous studies including a report by Giri et al. [26]. Grimes [27] has reported that in the absence of sunlight, the inactive vitamin D is diverted to the synthesis of cholesterol. Even so, intestinal absorption is the only source of fat-soluble vitamins and their mechanism of intestinal absorption remains unclear. Recent studies revealed the participation of several membrane proteins in cholesterol absorption evidenced by advancements in genome-editing, genome-wide association, and gene mutation analysis on cholesterol and intense studies in cholesterol absorption inhibitors. Interestingly, these analyses also revealed that cholesterol transporters can also transport fat-soluble vitamins [28]. In association with these studies, the present study identifies the serum levels of fat-soluble vitamins as effective markers of elevated cholesterol transportation and can be used as an early predictor of CVDs. It is a well-established fact that increased serum BCAAs were associated with elevated triglycerides and reduced HDL [29]. The present results also identify a similar pathology between serum HDL, triglycerides, and BCAAs. The BCAA in the present study exhibited a significant negative correlation with HDL and a strong positive correlation with triglycerides. Consumption of energy-dense and highly palatable foods has increased in recent years, and consumption of BCAAs is one such major behavioral change that received considerable attention in recent times. The most recent study by Latimer et al. [30] reported that an increase in BCAA consumption resulted in the dramatic growth of the heart and also increased the progression of cardiac diseases.

One of the main limitations of our study is that it includes data from free-living people with limited information on diet and lifestyle choices. The current study signifies the association of vital cardiovascular markers with a diverse class of micronutrients. Further research is required for a better understanding of the clinical significance of these micronutrients and also to understand the biochemical interference of vitamins, minerals, and amino acids in lipid metabolism.

## Conclusions

The current study examines the explicit correlation of micronutrients with various cardiovascular markers. Given the broad set of micronutrients evaluated, their significance in regulating vital cardiovascular markers was better understood. The study highlights the negative correlation of various vitamins and amino acids on cardiovascular health which was a significant observation. The deficiency of various micronutrients leads to a significant increase in cardiovascular disease risk due to the dysregulation of lipid and lipoprotein markers. Similarly, increased levels of certain micronutrients especially BCAAs lead to increased cardiovascular risk due to lipid and lipoprotein dysregulation. It is important to look at both deficiencies and overconsumption of micronutrients to optimize nutrient intake. The study suggests the proper monitoring of body vitals and optimal intake of micronutrients are to be considered for their effective roles in metabolic pathways to implement a risk reduction strategy for cardiovascular health.
